# Dysfunctional DNA Mismatch Repair Drives the Evolution of Gene Amplification in MTX-Resistant Human Colorectal Cancer Cells

**DOI:** 10.3390/ijms27093774

**Published:** 2026-04-23

**Authors:** Xu Wang, Siqing Li, Yanghe Liu, Yihan Gao, Xinyu Shi, Xuejian Han, Huishu Zhang, Xiangning Meng

**Affiliations:** 1 Department of Medical Genetics, School of Basic Medical Sciences, Harbin Medical University, Harbin 150081, China; 2 Key Laboratory of Preservation of Human Genetic Resources and Disease Control in China (Harbin Medical University), Ministry of Education, Harbin 150081, China; 3 Department of Microbiology, School of Basic Medical Sciences, Harbin Medical University, Harbin 150081, China; 4 Biotechnology Experimental Teaching Center, School of Basic Medical Sciences, Harbin Medical University, Harbin 150081, China

**Keywords:** gene amplification, ecDNAs, mismatch repair, DNA double strand breaks, MTX-resistance

## Abstract

Gene amplification resulting from double strand breaks (DSBs) is a typical genetic alteration in tumorigenesis and drug-resistant progression. Amplified oncogenes and drug-resistant genes are present on extrachromosomal DNAs (ecDNAs), or chromosomal homogeneously staining regions (HSRs). Considering the role of mismatch repair (MMR) as a sensor of DSBs, we hypothesized that MMR may be involved in gene amplification. We used two MTX-resistant HT-29 colorectal cancer cell lines, which served as models with amplified genes mainly in HSRs or ecDNAs. Expression of MSH2, a key protein in MMR, was increased following the acquisition of MTX-resistant. MMR inhibition was achieved by depleting MSH2. Suppression of MMR led to decreased copy numbers of amplified genes as well as the quantity of ecDNAs and HSR. This was caused by the decreased efficiency of DSBs repair, which resulted from the reduced ability of MMR to recruit DSBs repair proteins. Additionally, it accelerated the formation of micronuclei (MN)/nuclear buds (NBUDs), which functioned to eliminate the amplified genes. Furthermore, the suppression of MMR was capable of inhibiting cell proliferation and enhancing MTX-sensitivity in ecDNA-containing cells. Conversely, suppression of MMR had no effect on gene amplification in HSR-containing cells. Our findings demonstrate that MMR plays a pivotal role in gene amplification through mediating DSBs repair pathways and facilitating the formation of MN/NBUDs in ecDNA-containing cells. MMR is likely to emerge as a prime therapeutic target worthy of in-depth exploration in future clinical investigations.

## 1. Introduction

Tumorigenesis is often accompanied by genomic DNA copy number aberrations, especially gene amplification. DNA amplification is a molecular process that increases the copy number of a certain gene. It is also considered to play a crucial role in tumor drug resistance [1]. Intrachromosomal homogeneously staining regions (HSRs) and extrachromosomal DNAs (ecDNAs), which are alternatively referred to as double minutes (DMs), are the cytogenetic hallmarks of gene amplification [2]. ecDNAs are circular elements in nuclei and can replicate autonomously. Because ecDNAs lack a centromere, they segregate unequally into daughter cells during mitosis, which leads to more rapid cellular evolution and genetic variation [3,4]. ecDNAs represent an unstable form of gene amplification; Whereas, HSRs represent a stable form of amplification [5,6]. Previous studies have found that ecDNAs serve as the carriers of oncogenes or drug-resistant genes. However, the formation mechanism underlying the high copy numbers of these genes remains undetermined.

Hypotheses about the mechanisms of gene amplification include tandem duplication, the breakage–fusion–bridge (BFB) cycle, and chromothripsis [7,8]. Based on these hypotheses, ecDNAs and HSRs have always been thought to derive from DNA double-strand breaks (DSBs) in cells [9]. Cells utilize three major mechanisms to repair DSBs: homologous recombination (HR), classic non-homologous end joining (c-NHEJ) and alternative non-homologous end joining (a-NHEJ) [10,11]. Indeed, in our previous studies, we proposed that aberrant DSBs repair pathways, including HR and c-NHEJ could promote the formation of ecDNAs [12,13]. Moreover, we proposed that amplified genes can be eliminated outside primary nuclei through the formation of MN/NBUDs, which form during mitotic progression following DNA DSBs [14].

Mammalian DNA mismatch repair (MMR) is an evolutionarily conserved pathway primarily responsible for correcting base mismatches and insertion/deletion mismatches generated during DNA replication and recombination to maintain genome stability [15]. In the MMR pathway, MSH2 can form a MutSα heterodimer with MSH6 to recognize base–base and small insertion/deletion mismatches of one to about three extrahelical residues or form a MutSβ heterodimer with MSH3 recognizing insertion/deletion mismatches of two to about 10 extrahelical residues. After binding to mismatches, MutSα or MutSβ recruits downstream MLH1-PMS2 heterodimer to activate the repair process. Furthermore, Hong et al. demonstrated that MSH2, cooperating with MSH3 or MSH6, is recruited to the DSBs induced by laser micro-irradiation [16], indicating that MMR is essential for cellular response to DSBs. Studies have shown that MMR prevents recombination between heteroduplex containing mismatches and inhibits chromosomal translocation, deletion or insertion to maintain genome stability during HR [17,18]. Shahi and Smith et al. proposed that MMR participates in recruiting key c-NHEJ proteins (e.g., Ku70/Ku86), correcting and preventing the ligation of DNA ends with mismatches [19,20].

Although MMR proteins are known to recognize and promote the processing of aberrant or non-canonical structures in DSBs, whether MMR influences gene amplification arising from DSBs repair pathways, or participates in the formation of ecDNAs and HSRs remains unclear. In this study, we utilized two methotrexate (MTX)-resistant HT-29 colorectal cancer cell lines, which harbored amplified genes primarily in HSRs or ecDNAs as models, to explore the role of MMR in formation of ecDNAs and HSRs. Here, we report that MMR may be involved in gene amplification and drug resistance by mediating DSBs repair pathways and promoting MN/NBUDs formation in ecDNA-containing cells.

## 2. Results

### 2.1. MMR-Associated Proteins Contribute to the Development of MTX Resistance and Gene Amplification in HT-29 Cells

**Figure 1:**
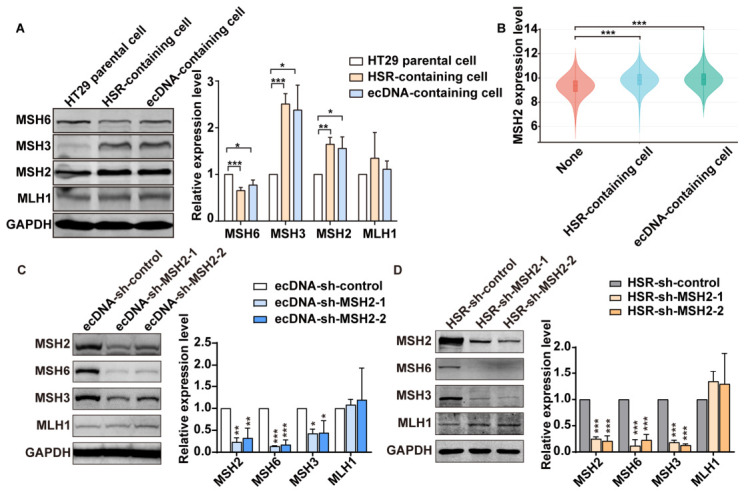
Dysfunctional mismatch repair (MMR) in cancer cells containing gene amplification. (**A**) In cells containing gene amplification, Western blot analysis reveals elevated MSH2 and MSH3 levels, whereas MSH6 is reduced. Right panel: densitometric analysis of protein expression normalized to GAPDH, comparing parental HT29 and MTX-resistant cells. (**B**) Bioinformatics analysis reveals elevated MSH2 mRNA levels in cancer cells containing gene amplification. (**C**,**D**) Western blot analysis of MSH2 and other key MMR proteins after MSH2 knockdown. Quantitative data were presented as mean ± SD (*n* = 3, * *p* < 0.05, ** *p* < 0.01, *** *p* < 0.001).

Previously, our group established MTX-resistant HT-29 colorectal cancer cell line (hereinafter referred to as MTX-resistant cells) harboring HSR or/and ecDNA. In cells resistant to 1.0 × 10^−5^ mol/L MTX, amplified genes localize within HSR, while in cells resistant to 1.0 × 10^−4^ mol/L MTX, amplified genes localize in both ecDNAs and HSR. To investigate whether MMR influences gene amplification-mediated drug resistance, we compared the expression levels of key MMR proteins (MSH6, MSH3, MSH2 and MLH1) between HT-29 parental cells and MTX-resistant cells. Western blot analysis revealed higher protein levels of MSH2 and MSH3, and lower MSH6 protein levels in MTX-resistant cells compared to parental cells (Figure 1A). MLH1 protein level had no significant change.

To verify the role of MMR in regulating gene amplification, we used the pan-cancer data from Hoon Kim et al. and the grouping based on gene amplification provided by the authors [21]. We analyzed the mRNA expression levels of MSH2, a key protein of the MMR system, in cancer cells without gene amplification, HSR-containing cancer cells, and ecDNA-containing cancer cells. The results of pan-cancer data analysis also demonstrated that the mRNA expression of MSH2 was significantly elevated in cancers with gene amplification (Figure 1B). These results suggest that MMR contributes to the development of MTX resistance and gene amplification.

MMR pathway can be suppressed by MSH2 functional deficiency [22]. To this end, we stably depleted MSH2 in ecDNA- and HSR-containing MTX-resistant cells, respectively, by shRNA transfection. We measured other key MMR proteins to verify MMR suppression. As expected, both MSH3 and MSH6 protein levels were significantly decreased, confirming the inhibition of MMR (Figure 1C,D).

### 2.2. MMR Inhibition Attenuates Gene Amplification in ecDNA-Containing MTX-Resistant Cells

**Figure 2:**
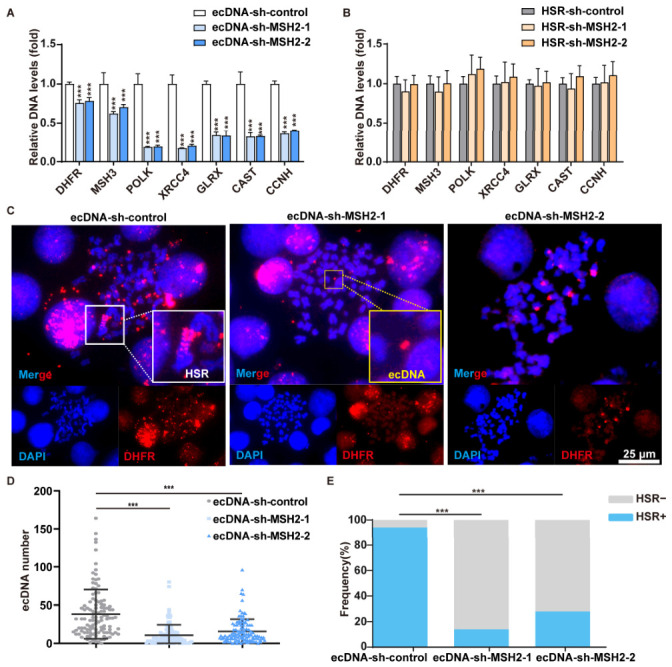
MMR inhibition attenuates gene amplification in ecDNA-containing cells, but not in those containing only HSR. (**A**,**B**) MSH2 depletion significantly decreased the amplification of *DHFR*, *MSH3*, *POLK*, *XRCC4*, *GLRX*, *CAST* and *CCNH* in ecDNA-containing cells but has no effect in HSR-containing cells, as determined by real-time PCR. Data are presented as mean ± SD (*n* = 3, *** *p* < 0.001). (**C**) FISH analysis of metaphase ecDNA-containing cells with a *DHFR* BAC probe. Red, *DHFR*; blue, DAPI-stained nuclei. ecDNA and HSR are indicated by yellow and white boxes, respectively. (**D**) Quantification of *DHFR*-amplified ecDNAs in control and MSH2-depleted cells. MSH2 depletion significantly reduced the number of such ecDNAs (*n* > 100, *** *p* < 0.001). (**E**) Quantification of *DHFR*-amplified HSR in control and MSH2-depleted cells. MSH2 depletion significantly reduced the number of such HSR (*n* = 50, *** *p* < 0.001).

Results from our prior comparative genomic hybridization (CGH) array revealed that *DHFR* and *MSH3* were amplified in both ecDNAs and the HSR, whereas *POLK*, *XRCC4*, *CCNH*, *GLRX* and *CAST* were only amplified within HSR on chromosome 5 in MTX-resistant cells [23]. To assess the effect of MMR suppression on gene amplification level, we evaluated the change in copy number. Real-time PCR analysis revealed that depletion of MSH2 in ecDNA-containing cells led to reduced amplification of genes located on both ecDNAs and the HSR (Figure 2A). By contrast, MMR inhibition showed no significant effect on gene amplification within the HSR in cells harboring only HSR. (Figure 2B). To further access the effect of MMR suppression on the form of gene amplification, fluorescence *in situ* hybridization (FISH) analysis was performed to label *DHFR*. Statistical analysis of the number of ecDNA and HSR in metaphase spreads, revealed that the average quantity of ecDNAs decreased by nearly 60%, and the number of karyotypes containing HSR decreased by more than 70% (Figure 2C–E). Consistent with the result of real-time PCR, in HSR-containing cells, MSH2 depletion had no effect on the form of gene amplification. Taken together, these results suggest that MMR may be involved in the formation of ecDNAs and HSR in ecDNA-containing cells but is unrelated to that in HSR-containing cells, which requires further investigation.

### 2.3. MMR May Be Involved in Formation of ecDNAs and HSR by Regulating DSBs Repair Pathways

**Figure 3:**
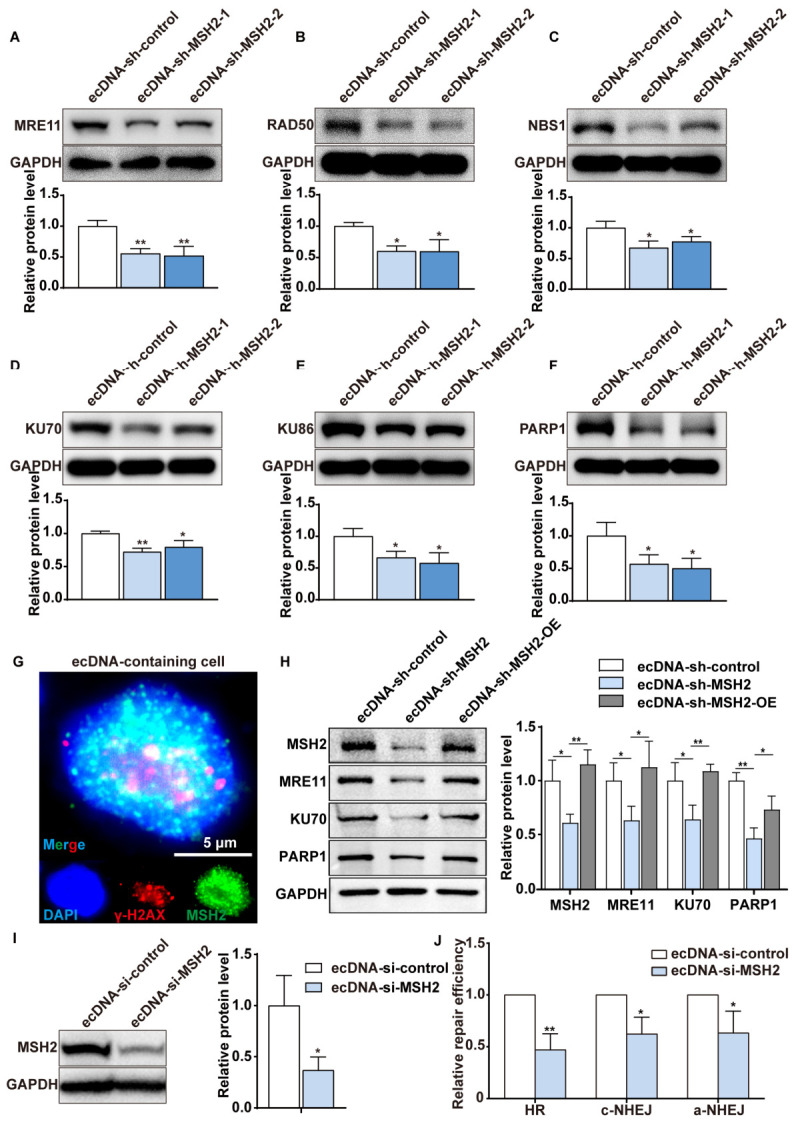
MMR may contribute to ecDNA and HSR formation via modulation of DSBs repair pathways. (**A**–**F**) Western blot analysis shows that depletion of MSH2 decreases the expression of key proteins involved in DSBs repair pathways (HR, c-NHEJ, and a-NHEJ). (**G**) Immunofluorescence images showing colocalization of MSH2 (green) and γ-H2AX (red) in the nuclei (blue) of ecDNA-containing cells. Scale bar, 5 μm. (**H**) Western blot analysis shows that rescue of MSH2 expression in MSH2-depleted cells restores the levels of key proteins involved in the HR, c-NHEJ, and a-NHEJ DSBs repair pathways. (**I**) Western blot analysis of MSH2 protein level in control and si-MSH2 transfected ecDNA-containing cells. (**J**) MSH2 knockdown reduces HR, c-NHEJ, and a-NHEJ repair efficiency in ecDNA-containing cells, as quantified by flow cytometry. Data are presented as mean ± SD (*n* = 3, * *p* < 0.05, ** *p* < 0.01).

Gene amplification may arise as a product of DSBs repair. Therefore, we hypothesize that MMR may be involved in ecDNA formation, possibly by regulating DSBs repair pathways. In HR pathway, MRN complex (comprising MRE11, RAD50 and NBS1) is recruited to DSBs to resect DNA ends and generate single-strand DNA. In c-NHEJ pathway, KU70 and KU86 form a heterodimer that recruits other key proteins to complete the repair process. However, PARP1 is a key protein in a-NHEJ pathway [24]. We measured the expression levels of the aforementioned proteins under treatment with 10^−3^ mol/L MTX to assess the capacity of the MMR pathway in repairing DNA double-strand breaks. As expected, the expression levels of key proteins in the DSBs repair pathways significantly decreased after MSH2 depleted (Figure 3A–F). Immunofluorescence analysis shows that MSH2 co-localizes with γ-H2AX in the nucleus of ecDNA-containing cells, further suggesting a potential link between MSH2 and the DNA damage response at sites of ecDNA formation (Figure 3G). Subsequently, we transfected MSH2-knockdown cells with an MSH2 expression plasmid to restore MSH2 level, generating ecDNA-shMSH2-OE cells. We then examined the expression of MRE11, KU70, and PARP1, which are key proteins in the HR, c-NHEJ, and a-NHEJ pathways, respectively. We found that restoration of MSH2 expression rescued the expression of these key proteins (Figure 3H). These results suggest that MSH2 knockdown sensitizes cells to MTX-induced DNA damage.

To further examine how MMR promotes DSBs repair, we employed integrated reporter assays for HR, c-NHEJ and a-NHEJ. In these chromosome-integrated reporters, functional green fluorescent protein (GFP) expression cassette can be restored only after I-SceI–induced DSBs are repaired by corresponding HR, c-NHEJ or a-NHEJ [25]. We respectively established ecDNA-containing MTX-resistant cell lines containing HR, c-NHEJ and a-NHEJ reporter. Functional GFP-expressing cells can be sorted by fluorescence-activated cell sorting (FACS) to evaluate DSBs repair efficiency. We depleted MSH2 by transfecting small interfering RNA (siRNA) against MSH2 (Figure 3I), and analyzed HR, c-NHEJ and a-NHEJ repair efficiencies in established reporter cell lines. A prominent attenuation in HR, c-NHEJ and a-NHEJ repair efficiencies was observed (Figure 3J). We concluded that MMR may facilitate ecDNAs and HSR formation by mediating HR, c-NHEJ and a-NHEJ repair pathways, which are involved in gene amplification.

### 2.4. MMR Inhibition Increases the Formation of MN/NBUDs Containing ecDNAs in ecDNA-Containing MTX-Resistant Cells

**Figure 4:**
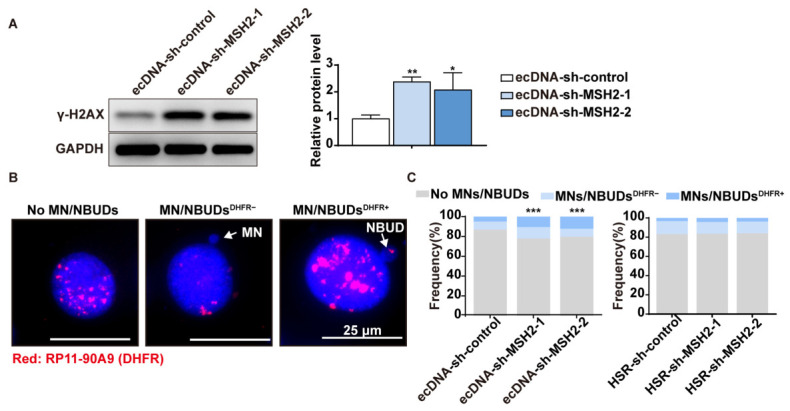
MMR suppression increases the formation of MN/NBUDs containing ecDNAs. (**A**) MSH2 knockdown significantly increased γ-H2AX protein levels in ecDNA-containing cells, as determined by Western blot. Data are presented as mean ± SD (*n* = 3, * *p* < 0.05, ** *p* < 0.01). (**B**) FISH analysis of interphase nuclei using a human *DHFR* BAC probe. Nuclei were categorized by MN status: without MN/NBUDs (**left**), with MN/NBUDs negative for *DHFR* signals (**middle**), and with *DHFR*-positive MN/NBUDs (**right**). Red, DHFR; blue, DAPI-stained nuclei. MN and NBUD are indicated by arrows. (**C**) Frequency of nuclei stratified by MN/NBUDs status. MSH2 knockdown increases MN/NBUDs formation in ecDNA-containing cells but has no effect in HSR-containing cells (*n* > 500, *** *p* < 0.001).

MN/NBUDs are generated in response to genotoxic agents and genetic instability. ecDNAs originating from DSBs can be packaged into NBUDs through nuclear envelope budding, eliminated via MN, and subsequently degradation by autophagy or cytoplasmic nucleases [26]. To this end, we detected the expression level of γ-H2AX, a sensitive molecular marker of DNA damage and repair. As expected, the protein level of γ-H2AX was significantly increased after MSH2 depletion (Figure 4A). To further access the effect of MMR on formation of MN/NBUDs, we performed FISH to analysis the number of MN/NBUDs after MSH2 depletion. MN/NBUDs scoring criteria: MNs were identified as small, round extra-nuclear bodies with DAPI intensity similar to that of the main nucleus and clearly separated from it. NBUDs were defined as protrusions from the main nucleus connected by a thin nucleoplasmic bridge, often exhibiting a “budding” morphology [27]. We captured over 500 nuclei and categorized them into three groups based on MN/NBUDs presence. Figure 4B shows representative nuclei without MN/NBUDs, with MN/NBUDs negative for amplified *DHFR*, and with MN/NBUDs positive for amplified *DHFR*. The presence of *DHFR* FISH signals within MN/NBUDs was scored only when the signal co-localized with the DAPI-stained MN/NBUDs structure. Statistical analysis revealed that MSH2 depletion increased the number of MN/NBUDs and significantly elevated the proportion of MN/NBUDs containing *DHFR* signals (Figure 4C). These results suggest a potential route for ecDNA loss under MMR deficiency.

### 2.5. MMR Inhibition Increases MTX-Sensitivity in ecDNA-Containing MTX-Resistant Cells

**Figure 5:**
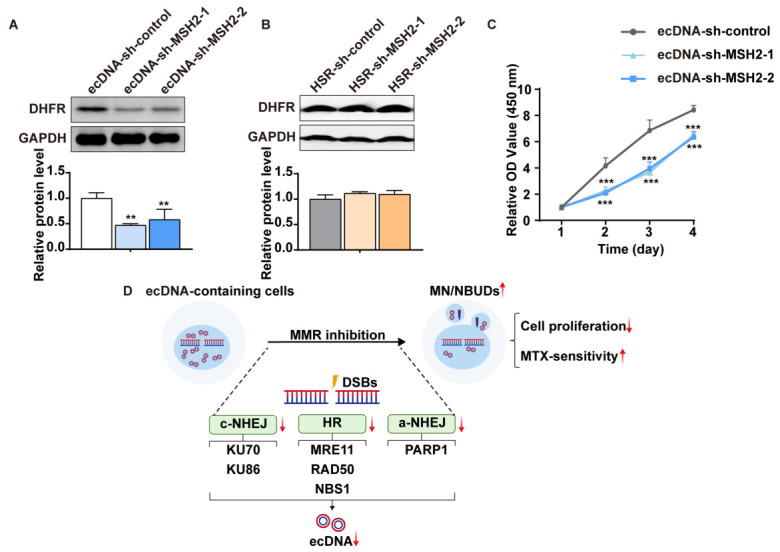
MMR suppression increases MTX-sensitivity in ecDNA-containing MTX-resistant cells. (**A**,**B**) Western blot analysis of DHFR protein level in ecDNA- and HSR-containing cells, with GAPDH serving as the loading control. MSH2 knockdown reduces DHFR expression in ecDNA-containing cells but has no effect in HSR-containing cells. Data are presented as mean ± SD (*n* = 3, ** *p* < 0.01). (**C**) Proliferation assay shows that MSH2 knockdown inhibits proliferation of ecDNA-containing cells. Data are presented as mean ± SD (*** *p* < 0.001). (**D**) Schematic diagram summarizing the proposed mechanism. Inhibition of MMR reduces gene amplification and MTX-resistance by attenuating DSBs repair pathways and accelerating MN/NBUDs formation in ecDNA-containing cells. Upward and downward red arrows indicate upregulation and downregulation, respectively. Abbreviations: MMR, mismatch repair; MN, micronuclei; NBUDs, nuclear buds; DSBs, double strand breaks; c-NHEJ, classic non-homologous end joining; HR, homologous recombination; a-NHEJ, alternative non-homologous end joining; ecDNA, extrachromosomal DNA.

MTX is an inhibitor of dihydrofolate-reductase (DHFR), a crucial enzyme in DNA biosynthesis. Overexpression of DHFR is a well-describe mechanism of MTX resistance. To assess the effect of MMR suppression on MTX resistance, we measured DHFR protein levels and half-maximal inhibitory concentration (IC_50_) values of cells. Western blot analysis revealed that DHFR protein level decreased following MSH2 depletion in ecDNA-containing cells but remained unchanged in HSR-containing cells (Figure 5A,B). IC_50_ values were calculated based on cell viability in response to different MTX concentrations. In ecDNA-containing cells, the IC_50_ value decreased by more than two-fold after MSH2 depletion, and a similar decrease was observed in HSR-containing cells (Table 1). Furthermore, proliferation assay demonstrated that MSH2 depletion suppressed cell proliferation in ecDNA-containing cells (Figure 5C). Therefore, we conclude that MMR inhibition sensitizes MTX-resistant cells to MTX.

## 3. Discussion

Gene amplification on ecDNAs or HSRs plays a critical role in accelerating cancer evolution and drug resistance. Although the nature of gene amplification in cancer remains incompletely understood, ecDNAs and HSRs are generally believed to originate from DSBs. Our previous studies have proved that two classical DSBs repair pathways, including HR and c-NHEJ are involved in ecDNAs formation [12,13]. Research has shown that components of DSBs repair are inactivated in MMR-deficient cell lines [28,29]. Kumar et al. demonstrated that MSH2-MSH3 heterodimer, a key factor in MMR, is required for binding to double-strand/single-strand junctions and initiating ATP-dependent repair to remove 3′ non-homologous tail during DSBs repair [30]. Although the role of MMR in DSBs repair has been recognized, its association with gene amplification remains underexplored. In this study, we observed a significant upregulation of MSH2 and MSH3 expression, and downregulation of MSH6 expression, in both HSR- and ecDNA-containing MTX-resistant cells. Despite the inconsistent expression changes in MMR proteins, these observations still raise the possibility that MMR may participate in gene amplification. The imbalanced MMR protein expression observed in MTX-resistant cells is mechanistically explained by the co-amplification of the *MSH3* and *DHFR* genes during the acquisition of MTX resistance, as they share a common promoter [23]. Overexpressed MSH3 sequesters MSH2 to form MutSβ heterodimers, leading to the degradation of MSH6 that is not incorporated into MutSα [31]. MutSβ primarily recognizes larger insertion-deletion loops, whereas MutSα is mainly involved in base–base mismatch repair and small loops. Therefore, ecDNA molecules, which are typically large and carry full-length oncogenes and regulatory elements [4], may be preferentially regulated by the MutSβ complex (MSH2–MSH3) rather than the MutSα complex (MSH2–MSH6).

To verify the association between MMR and gene amplification, we suppressed MMR by depleting MSH2 in ecDNA- and HSR-containing MTX-resistant cells respectively. We found a marked decrease in the protein level MSH3 and MSH6, indicating successful suppression of MMR. We then examined the effect of MMR on gene amplification in terms of both the level and the form of gene amplification. The results showed a dramatic decrease in the copy numbers of amplified genes, especially those localized within HSR in ecDNA-containing cells. FISH analysis of metaphase karyotypes demonstrated a decrease in counts of ecDNA and HSR in ecDNA-containing cells. The results indicate the critical role of MMR in formation of ecDNAs and HSR. However, MMR inhibition had no effect on HSR-containing cells. Studies suggest that the evolution of gene amplification during tumorigenesis may represent a single discontinuous event rather than a series of consecutive evolutionary steps [32]. We hypothesize that HSR formation may occur via multiple mechanisms, potentially dependent on the MTX concentration used to establish drug resistance. For example, HSRs and ecDNAs can arise from breakage–fusion–bridge (BFB) cycles between telomeres-lacking sister chromatids during replication; Gene amplification can also be generated by extrareplication and recombination, primary product in this model is ecDNAs derived from aberrant replication forks, and HSR subsequently formed by ecDNAs [2]. The molecular mechanisms underlying HSR formation in cell lines with varying degrees of MTX-resistance remain to be further investigated. Herein, we focus on the mechanisms of gene amplification in ecDNA-containing cells.

Studies have shown that ecDNAs clusters could form after DNA damage [33]. MMR has been found to be involved in HR and c-NHEJ to facilitate DSBs repair, beyond its role in post-replication repair. We performed DNA repair assays and found significant decreases in the repair efficiency of HR, c-NHEJ and a-NHEJ after MMR suppression. The results indicate that MMR may regulate gene amplification via mediating DSBs repair, including HR, c-NHEJ and a-NHEJ. This is supported by the finding that MMR removes mismatches during HR [34]. Moreover, studies have shown that mismatch repair promotes DNA end resection during HR and blocks polymerase theta-mediated end-joining [35]. In addition, Ucher et al. claim that MMR is required to generate DSBs that can be repaired by c-NHEJ, or to facilitate recruitment of c-NHEJ proteins [36]. Shahi et al. propose that MMR can regulate c-NHEJ through the association between MSH6 and KU70 [19]. Studies have also shown that MMR is important for class-switch recombination during antibody diversification via association with c-NHEJ and a-NHEJ [37,38]. In addition, EXO1, a key factor in DSBs repair, has been reported to interact with MSH2, providing another molecular link between the MMR and DSBs repair pathways [39]. Although, the study has shown that ecDNAs maintenance and replication were associated with other DNA repair pathways, such as base excision repair (BER) and nucleotide excision repair (NER) [40], it is important to note that the BER and NER pathways are primarily responsible for repairing small lesions on single-stranded DNA and helix-distorting damage. This is distinct from the process of ecDNAs formation, which involves large-scale chromosomal fragmentation and circularization. Taken together, our study proves that MMR may regulate ecDNAs formation by mediating HR, c-NHEJ and a-NHEJ in response to DSBs and proposes a novel mechanism for ecDNAs formation. In addition, we show that MMR inhibition may sensitize ecDNA-containing cells to DNA damage-inducing agents, suggesting a potential therapeutic vulnerability.

Our previous studies proved that inhibition of HR or c-NHEJ promotes the formation of MN/NBUDs to eliminate ecDNAs. To investigate the role of MMR in amplified genes elimination, we quantified the formation of MN/NBUDs and MN/NBUDs containing amplified genes. Statistical analysis indicated that MMR suppression increased the proportion of nuclei with MN/NBUDs and the frequency of MN/NBUDs containing amplified genes in ecDNA-containing cells. Beyond ecDNAs, studies show that damaged DNA fragments or even entire chromosomes can be eliminated from nuclei via MN/NBUDs [41]. Although the elimination of ecDNAs through MN/NBUDs has been reported by multiple research groups [7], whether HSR can be eliminated via MN/NBUDs remains unreported. Our results show that the number of karyotype-containing HSR decreased significantly in ecDNA-containing cells, suggesting that MMR suppression may also eliminate HSR via MN/NBUDs formation. Thus, we propose that MMR suppression promotes the formation of MN/NBUDs to eliminate ecDNAs and HSR.

From a clinical perspective, we measured the protein level of DHFR, the target of MTX, and IC_50_ values of the cells. We found that MMR suppression decreased DHFR protein levels in ecDNA-containing cells but had no effect on HSR-containing cells. Notably, both ecDNA- and HSR-containing cells exhibited increased sensitivity to MTX following MMR suppression. Clinical data indicate that gene amplification-driven drug resistance is a dynamic process involving multiple molecular events. Evidence also suggests that high-level clinical drug resistance is associated with gene amplification, whereas lower-level drug resistance may arise from other molecular mechanisms [42]. Although it is well known that MTX acts as a folate antagonist by competitively inhibiting DHFR activity, MTX is also capable of affecting various intracellular pathways independent of folate metabolism. For example, MTX can regulate oxidative stress, cell differentiation, anti-inflammation, DNA and protein demethylation, and protein acetylation, with effects depending on MTX concentration [43]. Therefore, we speculate that the sensitization effect in ecDNA-containing cells can be directly explained by reduced DHFR expression, the effect in HSR-containing cells likely involves the aforementioned, DHFR-independent pathways.

Several limitations of this study should be acknowledged. First, our findings are primarily based on in vitro cell line models; in vivo validation (e.g., animal models) has not yet been performed. Second, the dynamic processes of DNA damage/repair and MN/NBUDs formation following MMR inhibition have not been directly observed in real time or at the live-cell level. Third, while we demonstrate that MMR regulates ecDNA formation in ecDNA-containing cells, the mechanism underlying the unaltered gene amplification in HSR-containing cells remains to be elucidated.

In conclusion, our study identifies MMR as a novel regulator of gene amplification. We highlight that MMR is involved in DNA DSBs repair pathways to drive ecDNAs formation. Our work also reveals that MMR influences ecDNAs elimination by promoting MN/NBUDs formation. Reducing amplified drug-resistance genes via MMR suppression enhances cancer cells’ sensitivity to MTX (Figure 5D). Thus, we propose that MMR represents a potential therapeutic target for MTX-resistant cancers.

## 4. Materials and Methods

### 4.1. Cell Lines and Cell Culture

HT-29, human colorectal cancer cell line, purchased from cell bank of Chinese Academy of Sciences (Shanghai, China) was authenticated by the Beijing Microread Genetics (Beijing, China). HT-29 parental cells were grown in DMEM (GibcoBRL, Gaithersburg, MD, USA) supplemented with 15% fetal bovine serum (FBS, GibcoBRL). HT-29 MTX-resistant cell lines were established as described previously [23]. In brief, the culture medium was supplemented with 1.0 × 10^−7^ mol/L MTX as the initial drug concentration. After stable growth was achieved at this concentration, the MTX level was increased stepwise to a final concentration of 10^−4^ mol/L. HSR-containing and ecDNA-containing cells are resistant to 10^−5^ mol/L and 10^−4^ mol/L MTX, respectively.

### 4.2. Western Blot Analysis

Proteins from cell lysates were prepared, fractionated by SDS-PAGE, and transferred to PVDF membranes (Millipore, Burlington, MA, USA) as described previously [44]. Membranes were incubated with antibodies overnight at 4°C. The membrane was subsequently incubated with HRP-conjugated secondary antibody for 1 h at room temperature. Detection was achieved through enhanced chemiluminescence (ECL) using FluorChem R system (ProteinSimple, Santa Clara, CA, USA). Protein expression was quantified utilizing ImageJ software 1.46r. Detailed information on the antibodies is available in Supplementary Table S1.

### 4.3. RNA Interference and Plasmid Transfection

The shRNA lentivirus expression vector and the control vector (GeneCopoeia, Guangzhou, China) were respectively transfected into MTX-resistant HT-29 cells containing ecDNA or HSR with MOI = 10 according to the manufacturer’s protocol. The target sequences of shRNA for MSH2 were as follows: 5′-GCATCCAAGGAGAATGATTGG-3′, and 5′-GCAGCAAACTTACAAGATTGT-3′. 72 h after transfection, HSR-containing cells and ecDNA-containing cells were respectively selected with 0.5 μg/mL and 0.4 μg/mL puromycin to obtain stable transfected clones.

Transfection of HR, c-NHEJ and a-NHEJ reporter plasmids pHPRT-DRGFP, pimEJ5GFP and EJ2GFP-puro (AddGene, Cambridge, UK) into ecDNA-containing cells were performed using Lipofectamine2000 (Invitrogen, Carlsbad, CA, USA). Stable clones were selected by adding 0.4 μg/mL puromycin.

Transient transfections were performed using Lipofectamine2000 (Invitrogen, Carlsbad, CA, USA).

### 4.4. Real-Time PCR

Genomic DNA was prepared using QIAmp DNA Mini Kit (Qiagen, Dusseldorf, Germany) as described by the manufacturer’s protocol. Real-time PCR for a series of genes were performed using the Light Cycler 480 SYBRGreen Kit (Roche Applied Science, Mannheim, Germany). Thermocycling conditions (95 °C for 15 s, 60 °C for 30 s, 72 °C for 30 s and 40 cycles). Data were analyzed and presented relative to the expression level of ACTB. 2^−ΔΔCt^ quantification was performed as previously described [45]. The DNA primers are available in Supplementary Table S2.

### 4.5. Fluorescence In Situ Hybridization (FISH)

Cells were synchronized in metaphase with 0.2 μg/mL colcemid (Sigma-Aldrich Co. LLC, St. Louis, MO, USA) for 1 h, collected by trypsinization and centrifuged. After hypotonic treatment and fixation, cells were dropped on a superfrost microscope slide. FISH probe for *DHFR* gene was synthesized by BAC cloning PR11-90A9 and were labeled with Cy3-dUTP (Invitrogen, Carlsbad, CA, USA) [23]. Hybridization of FISH probes to interphase and metaphase spreads of cells as described in previously described [12]. Photographs were captured using a fluorescence microscope (Leica Microsystems, Solms, Germany) at 1000× magnification, and acquired images were merged by MetaMorph Imaging System (Leica Microsystems, Solms, Germany). ecDNAs were identified by the presence of multiple, paired, small, dot-like extrachromosomal signals. HSR was defined as large, homogeneously stained chromosomal region integrated within a chromosome arm [46]. All images were independently evaluated by two researchers to ensure consistent classification. For karyotype analysis, more than 100 metaphase cells were examined for ecDNAs, and 50 metaphase cells were examined for HSR. For micronucleus analysis, more than 400 interphase cells were scored.

### 4.6. Immunofluorescence

Cells were fixed with 4% paraformaldehyde for 15 min, permeabilized with 0.2% Triton X-100 for 10 min, and blocked with 5% goat serum for 1 h at room temperature. Primary antibodies against MSH2 (dilution 1:100, Cat # 33-7900) (Invitrogen, Carlsbad, CA, USA) and γ-H2AX (dilution 1:50, Cat # 05-636-I) (Millipore, Burlington, MA, USA) were incubated overnight at 4 °C, followed by Alexa Fluor-conjugated secondary antibodies (dilution 1:500) (Abbkine Scientific Co., Ltd., Wuhan, China) for 1 h at room temperature. Nuclei were stained with DAPI. Images were acquired using a fluorescence microscope (Leica Microsystems, Solms, Germany).

### 4.7. HR, c-NHEJ and a-NHEJ Assays

The DNA repair assay was performed as described in a previous study [25]. Briefly, siRNA targeting MSH2 (or control siRNA) and the I-SceI expression plasmid (Addgene, Cambridge, UK) were co-transfected into cells stably expressing the HR, c-NHEJ, or a-NHEJ reporter plasmids. Cells carrying the reporter construct but not transfected with the I-SceI plasmid served as a negative control to determine background GFP fluorescence. Three days after transfection, GFP fluorescence was measured by flow cytometry (BD Bioscience, San Jose, CA, USA). A minimum of 30,000 events were collected per sample. The gating strategy was based on forward/side scatter to exclude debris and doublets, and data were analyzed using BD Diva software (v8.0.1). The percentage of GFP-positive cells was corrected by subtracting the background signal obtained from the negative control.

### 4.8. Proliferation Assay

Cells in logarithmic growth phase were seeded into 96-well plates at a concentration of 1000 cells/well. Cell proliferation was detected at the indicated time points using a CCK8 kit (Biosharp Life Sciences, Beijing, China) following the manufacturer’s instructions. The optical density (OD) value at 450 nm wavelength was measured by a microplate reader and the cell proliferation curve was analyzed.

### 4.9. Drug Sensitivity Assay

Cells in logarithmic growth phase were seeded into 96-well plates at a concentration of 5000 cells/well and were treated with MTX at various concentrations (from 2.75 × 10^−2^ mol/L to 1.68 × 10^−6^ mol/L, four-fold serial dilutions) for 72 h. At the end of the treatment, the medium was replaced with mixture of 100 μL medium and 20 μL CellTiter 96 AQueous One Solution Cell Proliferation Assay (Promega, WI, USA). Cell viability was determined by measuring the absorbance at 492 nm using a microplate reader (Tecan Austria GmbH, Grödig, Austria). Based on the absorbance values of control wells (untreated with MTX) and MTX-treated wells, the cellular growth inhibition rate at each MTX concentration was calculated, and IC_50_ values were subsequently determined from these inhibition rates using the Improved Karber method [47].

### 4.10. Statistical Analysis

All experiments were repeated more than three times. Statistical analysis for experimental results of Western blot, real-time PCR, change in the ecDNA number, DSBs repair assays, cell proliferation and drug sensitivity assays between 2 groups were used unpaired Student’s *t* test, one- or two-way analysis of variance (ANOVA) followed by the Dunnett’s test used for comparing more than 2 groups. The statistical significance of differences for HSR and micronucleus effluents was analyzed by Chi-square analysis. *p* less than 0.05 were considered statistically significant.

## Figures and Tables

**Table 1:** MTX sensitivity (IC_50_) of ecDNA- and HSR-containing cells.

HT-29 Cell Line	IC_50_ (mol/L)	Fold Change
ecDNA-sh-control	3.35 × 10^−4^ ± 4.58 × 10^−5^	1
ecDNA-sh-MSH2-1	1.40 × 10^−4^ ± 2.89 × 10^−5^	2.39 ** vs. ecDNA-sh-control
ecDNA-sh-MSH2-2	1.50 × 10^−4^ ± 4.21 × 10^−5^	2.24 ** vs. ecDNA-sh-control
HSR-sh-control	2.69 × 10^−4^ ± 7.89 × 10^−6^	1
HSR-sh-MSH2-1	1.50 × 10^−4^ ± 1.79 × 10^−6^	1.79 *** vs. HSR-sh-control
HSR-sh-MSH2-2	1.28 × 10^−4^ ± 2.45 × 10^−6^	2.10 *** vs. HSR-sh-control

*n* = 3, ** *p* < 0.01, *** *p* < 0.001.

## Data Availability

The original contributions presented in this study are included in the article/Supplementary Materials. Further inquiries can be directed to the corresponding authors.

## Supplementary Materials

The following supporting information can be downloaded at: https://www.mdpi.com/article/10.3390/ijms27093774/s1.

## Abbreviations

The following abbreviations are used in this manuscript:

DSBs	Double strand breaks
ecDNAs	Extrachromosomal DNAs
HSR	Homogeneously staining regions
MMR	Mismatch repair
MN	Micronuclei
NBUDs	Nuclear buds
BFB	Breakage–fusion–bridge
HR	Homologous recombination
c-NHEJ	Classic non-homologous end joining
a-NHEJ	Alternative non-homologous end joining
MTX	Methotrexate
CGH	Comparative genomic hybridization
FISH	Fluorescence in situ hybridization
GFP	Green fluorescent protein
FACS	Fluorescence-activated cell sorting
siRNA	Small interfering RNA
DHFR	Dihydrofolate-reductase
BER	Base excision repair
NER	Nucleotide excision repair
