# Supplementary material for: Dysfunctional DNA Mismatch Repair Drives the Evolution of Gene Amplification in MTX-Resistant Human Colorectal Cancer Cells
Source: Int J Mol Sci. 2026 Apr 23;27(9):3774. doi: 10.3390/ijms27093774 (PMC13163856; doi:10.3390/ijms27093774)
Supplement: Supplementary file 1 [file ijms-27-03774-s001.zip › ijms-4229272-supplementary.pdf]

Table S1. Detailed antibody information

Antibody	Company	Catalog number	Concentration
MSH6	BD Transduction Laboratories	611390	1:250
MSH3	BD Transduction Laboratories	610918	1:1000
MLH1	BD Transduction Laboratories	554073	1:500
MSH2	Invitrogen	33-7900	1:250
MRE11	GeneTex	GTX70212	1:500
RAD50	Upstate	05-525	1:1000
NBS1	Abnova	PAB12377	1:1000
KU70	Abcam	ab92450	1:1000
KU86	Santa Cruz Biotechnology Inc	sc-1484	1:100
PARP1	Santa Cruz Biotechnology Inc	sc-25780	1:100
γ-H2AX	Millipore	05-636-I	1:500
DHFR	Abnova	MAB22083	1:1000
GAPDH	Kang Chen Bio-tech	KC-5G4	1:5000

Table S2. Primers used in this study

Primer name	Sequence
DHFR-F	ATTTTGTTCAGTGCCTACCACA
DHFR-R	GCCTGAATGATATCTACAAGCTG
MSH3-F	TGTCTGGTGTTTCGCCTGAT
MSH3-R	TTAGCCAATAACCGCTCTAC
CCNH-F	GTATTGCAGCACTGATTATGTCC
CCNH-R	TCATGAAAATAGCCATAGGTGA
GLRX-F	CCCACATTGTAGGGAATCAT
GLRX-R	CCCACAGTCTATTCGTAGCA
CAST-F	TTGACTCCATAGCCAACCTT
CAST-R	GTCACTTTTCCCAGAATCCG
ACTB -F	CTTCTACAATGAGCTGCGTG
ACTB -R	AAGCAAATAGAACCTGCAGAG
